# An Assessment of Epidemiology Capacity in a One Health Team at the Provincial Level in Thailand

**DOI:** 10.3390/vetsci3040030

**Published:** 2016-10-17

**Authors:** Soawapak Hinjoy, Arthicha Wongkumma, Somkid Kongyu, Punnarai Smithsuwan, Paphanij Suangtho, Thitipong Yingyong, Sunicha Chanvatik, Soledad Colombe

**Affiliations:** 1 Department of Disease Control, Bureau of Epidemiology, Active Surveillance Section, Nonthaburi 11000, Thailand; tsuwan1@hotmail.com (A.W.); skongyu@gmail.com (S.K.); smithsuwan@gmail.com (Pu.S.); paphanij@gmail.com (Pa.S.); thity_24@yahoo.com (T.Y.); 2 Thai Coordinating Unit for One Health, Nonthaburi 11000, Thailand; sunicha.c@gmail.com; 3 Center for Global Health, Weill-Cornell Medical College, 1300 York Ave, New York, NY 10065, USA; soledad.colombe@gmail.com

**Keywords:** assessment, epidemiology capacity, One Health approach

## Abstract

A multi-sectoral core epidemiology capacity assessment was conducted in provinces that implemented One Health services in order to assess the efficacy of a One Health approach in Thailand. In order to conduct the assessment, four provinces were randomly selected as a study group from a total of 19 Thai provinces that are currently using a One Health approach. As a control group, four additional provinces that never implemented a One Health approach were also sampled. The provincial officers were interviewed on the epidemiologic capacity of their respective provinces. The average score of epidemiologic capacity in the provinces implementing the One Health approach was 66.45%, while the provinces that did not implement this approach earned a score of 54.61%. The epidemiologic capacity of surveillance systems in provinces that utilized the One Health approach earned higher scores in comparison to provinces that did not implement the approach (75.00% vs. 53.13%, *p*-value 0.13). Although none of the capacity evaluations showed significant differences between the two groups, we found evidence that provinces implementing the One Health approach gained higher scores in both surveillance and outbreak investigation capacities. This may be explained by more efficient capacity when using a One Health approach, specifically in preventing, protecting, and responding to threats in local communities.

## 1. Introduction

Recent environmental climate change and natural disasters have increased the number of health issues and diseases across Thailand. Both newly emerging and re-emerging diseases have led to a substantial impact on human health and the economy. The increased disease burden affects not only individuals, but Thai society as a whole, hindering development opportunities in resource limited areas. The outbreak of Avian Influenza in Thailand during 2004 and 2006 spread widely across the country, resulting in the destruction of an estimated 65 million birds, and expenses of more than $35 million USD of public-money used as compensation to the bird owners affected [1,2]. Modern public health practice calls for a comprehensive, interdisciplinary approach to disease prevention and control, embracing not only the principles of public health, but also veterinary medicine and ecosystem health, along with other disciplines. This new concept of public health practice is known as the One Health approach. It has been adopted with great enthusiasm by the veterinary profession and by the international agencies in charge of the control of zoonotic diseases, most notably the Food and Agriculture Organization (FAO), the World Health Organization (WHO), and the World Organization for Animal Health (OIE). The goal of One Health is to address such issues as food safety, food security, antimicrobial resistance, climate change, and the human–animal bond from a diverse, multidisciplinary perspective [3]. During the avian influenza outbreak, the Thai operation teams were renamed as the Surveillance and Rapid Response Team, or SRRT, and a target was set for each district in Thailand to have at least one team and one provincial SRRT in every province, including Bangkok [4]. In 2012, a short course focusing on basic epidemiology and event-based surveillance was developed to train SRRT personnel in communities around Thailand. The course was specifically directed at public health volunteers [5].

Part of the Thai Ministry of Public Health, the Bureau of Epidemiology, Department of Disease Control, has strengthened the surveillance and investigation of zoonotic diseases at the provincial and district level through the “One Health Epidemiology Teams at Provincial Level” Project. This was originally accomplished with the technical and financial support from the United States Agency for International Development (USAID) and the United States Centers for Disease Control and Prevention (U.S. CDC). After the program’s initial implementation in 2012, Thailand continued to independently strengthen the One Health epidemiology effort by expanding team coverage to 19 provinces by 2016. This project accomplished the formation of provincial One Health epidemiology teams and professional networks by uniting various professionals such as medical doctors, veterinarian officers, nurses, public health officers, local authorities, and officers who were already operating in the same province. This pilot project was a collaborative effort among the Department of Disease Control, the Department of Livestock Development, the Department of Natural Park, the Zoological Park Organization of Thailand, the Thailand Field Epidemiology Training Program (FETP)/FETP-Veterinarians (FETP-V), USAID, U.S. CDC, universities, and other partners. This project aimed to strengthen Thailand’s emerging infectious diseases (EIDs) preparedness and response capacity, specifically focusing on disease surveillance and outbreak investigation at the district or provincial level. Activities included a team-building workshop, a one-week workshop for employees from multiple sectors to work together and develop a five-month One Health field project, and a final seminar, where findings, experiences, and lessons learned from the field projects were presented and shared among the stakeholders. A cross-sectional study was conducted to assess public health teams’ capacity in three essential services of epidemiology when comparing provinces implementing a One Health approach to provinces not implementing it. The objective of this study was to measure the One Health teams’ capacity in three essential services of epidemiology, including (1) surveillance—the ability to detect health threats—(2) data reporting—the ability to report health threat—and (3) outbreak investigation—the ability to conduct epidemiological investigations and control the spread of disease at the source of infection.

## 2. Materials and Methods

**Figure 1:**
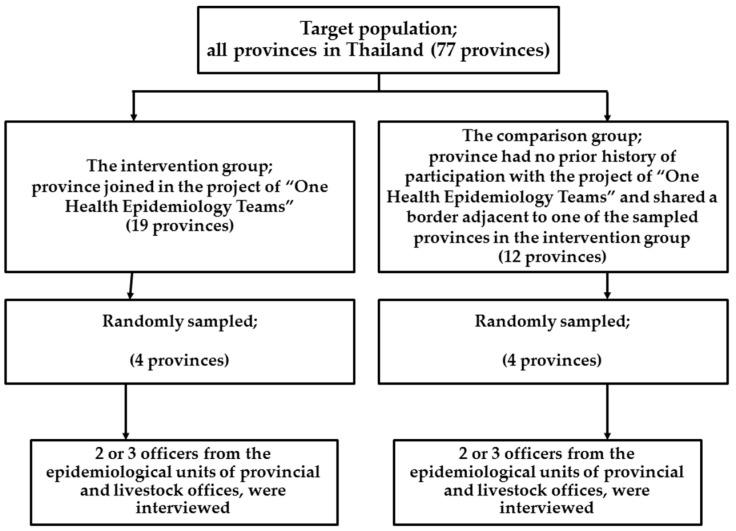
Flow chart of study population: assessment of epidemiology capacity in One Health teams at the provincial level in Thailand.

The pilot project for the establishment of “One Health Epidemiology Teams at the Provincial Level” included few provinces (19 provinces). We used a simple random sampling technique to select four provinces from this initial group entitled “the intervention group” without the calculation of sample sizes due to budget limitations. The comparison group consisted of four provinces that were randomly selected by using a 1 (Intervention group) to 1 (Comparison group) ratio. For the comparison group, the sampling frame came from a list of 12 provinces that shared a border adjacent to one of the sampled provinces in the intervention group. The inclusion criterion for the comparison group required that the province had no prior history of participation with the “One Health epidemiology teams at provincial level” project (Figure 1).

Measurements of the three essential services of epidemiology mentioned above were conducted in both the intervention group and the comparison group.

A letter was sent to all eligible provinces from the random sampling inviting the provinces to participate in the study and providing information on the requirements. This cross-sectional study was conducted from March to June 2016. After gaining permission from the directors of provincial public health and livestock offices to conduct the study, two or three officers from each disease control unit were asked to answer a standardized questionnaire. Officers were chosen from the epidemiological units of provincial public health offices, and the animal health and sanitary units of provincial livestock offices. Furthermore, a person-to-person interview was conducted by trained officers from the Bureau of Epidemiology.

Characteristics of provinces were also recorded using a standardized questionnaire, including population density, living conditions of livestock farmers, and average income of population. Information about the capacities of epidemiological teams focusing on zoonotic diseases was also collected, including detection of health threats, reporting of health threats, outbreak investigation, and determination of control measures. There were 18 questions to explore the three epidemiology services capacities divided into seven questions for assessment of surveillance capacities (the score was between 0 and 7), four questions for assessment of data reporting (the score was between 0 and 4) and seven questions for assessment of outbreak investigation capacities (the score was between 0 and 7). A full score of 1 was awarded per question if both public health and livestock officers could show the documented evidence to the interviewers of the Bureau of Epidemiology. A half score of 0.5 was given if there was documented evidence from only one sector. Zero meant there was a lack of documents in both sectors.

Data management and all analyses were performed using SPSS version 20.0 (SPSS, Inc., Chicago, IL, USA). Scoring of the three essential services of epidemiology was treated as a continuous variable. Simple tabulation was used to describe the data by using independent sample *t* test. A two-sided *p*-value < 0.05 was considered statistically significant in all analyses.

## 3. Results

There were no significant differences in the general characteristics between provinces implementing the One Health approach and provinces that did not (Table 1).

The average score from the assessment of the three essential services of epidemiology in provinces implementing the One Health approach was 66.45% (range 50.00%–78.95%), while the average score of provinces that did not implement One Health approach was 54.61% (range 36.84%–68.42%). There was no significant difference in the average scores between the two groups.

We found higher scores in the intervention group than in the comparison group for the following areas of surveillance capacities: the core capacities of determining priority diseases in the area, the capability of raising awareness when abnormal event occurred, presenting visualization of diseases as a spot map, sharing database surveillance, getting members of both human health and animal health sectors trained in surveillance, and building participating communities to the loop of surveillance network (Table 2). However, there were no statistically significant differences for any of the core capacities of surveillance between provinces implementing the One Health approach and provinces not implementing the approach.

In the area of data reporting, all provinces (100%) implementing the One Health approach had an established reporting system and flow of communication among human health, animal health, and local administrations. The provinces that did not implement the One Health approach had higher scores in logging the reporting system and record keeping when compared with the provinces that implemented the One Health approach (Table 3).

There were not many differences in areas of outbreak investigation between the two groups. We found higher scores in the intervention group than in the comparison group for the following areas of outbreak investigation: preparedness for joint outbreak investigation, creation of guidelines for the investigation and control of disease outbreaks, readiness of supplies and equipment for the joint investigation team, and training in samples’ collection (Table 4).

In conclusion, the capacity of surveillance systems in the intervention group had much higher scores than the comparison group (75.00% vs. 53.13%). Similar results were shown in regard to the capacity of outbreak investigation (60.71% vs. 50.00%). However, there were no statistically significant differences (*p*-value 0.13 and *p*-value 0.36, respectively). Furthermore, provinces implementing the One Health approach gained lower scores in data reporting capacity when compared to provinces that did not implement the One Health approach (59.38% vs. 65.63%). There was again no statistically significant difference between the two groups (*p*-value 0.60).

## 4. Discussion

Recent EIDs, such as Avian Influenza H5N1, Ebola, West Nile Virus, and Nipah Virus exemplify the integral and dangerous interconnection of animal and human health. About 70% to 80% of EIDs in humans have originated from animals [6]. Economic growth, changes in habitation and farming systems, deforestation, increased travel and trade, and climate change are important factors contributing to the spread of infectious diseases. Improved infectious disease prevention and management can be accomplished through close collaboration across sectors, including human, animal, and environmental health, along with other disciplines, including the natural and social sciences. Many countries adopted the One Health approach as a key factor in confronting the threat of EIDs. Some examples include the establishment of a One Health office in Kenya [7], and the improved control of rabies in Bali, hydatid disease in Nepal, Q-fever in the Netherlands, and food-borne Salmonella in the European Union (EU) [8].

However, our study was unable to show any significant evidence of how the One Health approach might bring benefits to the community. This non-significant result was most likely due to small sample sizes driving a lack of statistical power in the study. Throughout its long history, epidemiology has been utilized in a multitude of different ways. One important factor, originally demonstrated by Morris [9], was the use of evidence as an estimation of the magnitude of disease risk within a population and the projection of that risk into the future. The European Centre for Disease Control (ECDC) has created a list of core competencies for public health epidemiologists working in the area of communicable disease surveillance and response. Some of these core competencies were designed specifically for epidemiological surveillance and field investigation, and emphasize the capability of defining health measures to control disease outbreaks [10].

Provinces implementing a One Health approach showed higher epidemiologic capacity scores for surveillance and outbreak investigation than provinces that did not utilize the One Health approach (Table 2 and Table 4). In order to strengthen surveillance capacity (Table 2), provincial One Health teams established a database allowing multiple sectors to share surveillance information. Sane and Edelstein [11] reviewed the benefits of surveillance data sharing for the WHO’s Global Influenza Surveillance and Response System, which resulted in mandates improving health and lowering costs for society as a whole and activities of data sharing also combined with a mandate from revision of the International Health Regulations and Global Health Security Agenda [12]. Another important factor of surveillance capacity that could greatly benefit communities is the empowering of disease surveillance, control, and prevention in local communities. Increasing awareness among rural communities could help in preventing zoonotic disease. Some examples of successful community empowerment include the prevention of rabies in Bali [13] and community-based active surveillance provided a potential cost-effective strategy for improving estimates of rabies incidence and epidemiology to inform veterinary and policy decision-making in Kenya [14].

Preparedness of joint epidemiological investigation in promptly responding to abnormal events was analyzed using investigation reports (Table 4). The reports showed evidence that both human and animal health investigation teams could be combined and work together following all national guidelines for priority diseases criteria. The merging of these investigation teams showed a great benefit in stopping the spread of disease and preventing the spillover of diseases into humans. When an outbreak is identified and linked to a specific site, an immediate response is critical to control the source of infection [15]. The epidemiologic capacity of data documentation in provinces implementing One Health approach was weaker than provinces without the One Health approach (Table 3). This weakness most likely comes from a higher usage of internal communication among personnel such as social media, increasing unofficial documentation. While interviewing provincial officers, it was observed that some officers could not find a record of notification about abnormal events from partners. However, the officers did show how they communicated and notified each other across multiple sectors about abnormal events using social networks such as Line app and Facebook. This theory is further supported by the evidence brought by the 19 initial intervention provinces, which had a list of networks with names, telephone numbers, addresses, e-mail addresses from the human health, animal health, and local administrations sectors. This capacity is shown in Table 3 as a capacity of establishment of a reporting system and flow of communication among parties.

## 5. Conclusions

No significant differences were observed for the epidemiologic capacities in the three essential services of epidemiology between the provinces implementing a One Health approach and the provinces that did not implement it. Due to sample size limitations, we could make conclusions about the impact of implementing a One Health approach on providing epidemiological services to communities in Thailand. However, the average epidemiologic capacity scores for surveillance and outbreak investigation in the provinces implementing a One Health approach were higher than those that did not implement a One Health approach, which shows a potential benefit to implementing a One Health approach. The One Health approach could thus be used to increase capacity development and to better prevent, protect, and respond to any threats in these communities.

## Figures and Tables

**Table 1:** Population characteristics between provinces that implemented the One Health approach and provinces that did not, Thailand 2016.

No.	Characteristics of Provinces	Provinces Implementing One Health Approach (Mean Scores ± Standard Deviations)		*p*-Value
		Yes (*n* = 4 Provinces)	No (*n* = 4 Provinces)	
1	Population density per km^2^	94.12 ± 42.91	122.38 ± 44.53	0.40
2	Number of households of livestock farmers	46,949.00 ± 37,235.17	67,047.00 ± 26,460.56	0.41
3	Average income per capita (Baht)	69,586.75 ± 11,670.20	70,467.00 ± 16,439.27	0.93

**Table 2:** Assessment of surveillance capacities between the provinces that implemented the One Health approach and provinces that did not implement the One Health approach in Thailand, 2016.

No.	Core Capacities of Surveillance Abilities	Provinces Implementing One Health Approach (Mean Scores ± Standard Deviations)		*p*-Value
		Yes (*n* = 4 Provinces)	No (*n* = 4 Provinces)	
1	Both sectors had mutual consideration to set priority diseases in the province	0.75 ± 0.29	0.75 ± 0.29	1.00
2	Establishment of “alert” and “epidemic” threshold values for diseases with epidemic tendencies	0.63 ± 0.25	0.38 ± 0.48	0.39
3	Presence of risk mapping to present high-risk areas	0.63 ± 0.48	0.50 ± 0.58	0.75
4	Sharing surveillance databases with relevant organizations	0.88 ± 0.25	0.75 ± 0.29	0.54
5	Training of members of SRRT in the district level in surveillance methodology	0.88 ± 0.25	0.75 ± 0.29	0.54
6	Training of members of SRRT in public health emergencies of international concern	0.50 ± 0.40	0.50 ± 0.00	1.00
7	Building empowering surveillance, control and prevention in local communities	0.75 ± 0.29	0.63 ± 0.25	0.54
Mean total score, Standard deviation		75.00% ± 18.40%	53.13% ± 16.53%	0.13

**Table 3:** Assessment of data report capacities between the provinces implementing One Health approach and not implemented One Health approach in Thailand, 2016.

No.	Core Capacities of Documented Data Report Abilities	Provinces Implementing One Health Approach (Mean Scores ± Standard Deviations)		*p*-Value
		Yes (*n* = 4 Provinces)	No (*n* = 4 Provinces)	
1	Establishment of reporting system and flow of communication among human health, animal health and local administrations	1.00 ± 0.00	0.88 ± 0.25	0.36
2	Regular reporting to other partners	0.75 ± 0.29	0.63 ± 0.25	0.54
3	Creating Standard Operating Procedure for reporting	0.25 ± 0.29	0.38 ± 0.25	0.54
4	Standardized incident reporting form	0.38 ± 0.25	0.75 ± 0.29	0.10
Mean total score, Standard deviation		59.38% ± 15.73%	65.63% ± 15.73%	0.60

**Table 4:** Assessment of outbreak investigation capacities between the provinces implementing One Health approach and the provinces did not implement One Health approach in Thailand, 2016.

No.	Core Capacities of Outbreak Investigation	Provinces Implementing One Health Approach (Mean Scores ± Standard Deviations)		*p*-Value
		Yes (*n* = 4 Provinces)	No (*n* = 4 Provinces)	
1	Preparedness for joint epidemiological investigation to promptly respond to abnormal events	0.75 ± 0.29	0.50 ± 0	0.13
2	List of rapid response team members with contact numbers in both human and animal health sectors	0.63 ± 0.25	0.63 ± 0.25	1.00
3	Training of SRRT members in field epidemiology	0.63 ± 0.25	0.63 ± 0.25	1.00
4	Creation of guidelines for the investigation and control of disease outbreaks	0.63 ± 0.25	0.38 ± 0.25	0.21
5	Preparedness of supplies or equipment if outbreak occurred	0.50 ± 0	0.38 ± 0.25	0.36
6	Preparedness of personal protective equipment if outbreak occurred	0.50 ± 0	0.50 ± 0	1.00
7	Training of SRRT members in samples’ collection	0.63 ± 0.25	0.50 ± 0.41	0.62
Mean total score, Standard deviation		60.71% ± 7.14%	50.00% ± 20.20%	0.36
